# Multi-walled carbon nanotube oxidation dependent keratinocyte cytotoxicity and skin inflammation

**DOI:** 10.1186/s12989-018-0285-x

**Published:** 2019-01-08

**Authors:** Brian C. Palmer, Sarah J. Phelan-Dickenson, Lisa A. DeLouise

**Affiliations:** 1 Department of Environmental Medicine, University of Rochester Medical Center, New York, USA; 2 Department of Biomedical Engineering, University of Rochester, Rochester, NY USA; 3 Department of Dermatology, University of Rochester Medical Center, Rochester, NY USA; 4 University of Rochester Medical Center, School of Medicine and Dentistry, 601 Elmwood Avenue, Box 697, Rochester, NY 14642 USA

**Keywords:** Nanoparticle, Carbon nanotube, Skin, Allergy, Dermatitis

## Abstract

**Background:**

The effects of carbon nanotubes on skin toxicity have not been extensively studied; however, our lab has previously shown that a carboxylated multi-walled carbon nanotube (MWCNT) exacerbates the 2, 4-dinitrofluorobenzene induced contact hypersensitivity response in mice. Here we examine the role of carboxylation in MWCNT skin toxicity.

**Results:**

MWCNTs were analyzed by transmission electron microscopy, zetasizer, and x-ray photoelectron spectroscopy to fully characterize the physical properties. Two MWCNTs with different levels of surface carboxylation were chosen for further testing. The MWCNTs with a high level of carboxylation displayed increased cytotoxicity in a HaCaT keratinocyte cell line, compared to the MWCNTs with intermediate levels of carboxylation. However, neither functionalized MWCNT increased the level of in vitro reactive oxygen species suggesting an alternative mechanism of cytotoxicity. Each MWCNT was tested in the contact hypersensitivity model, and only the MWCNTs with greater than 20% surface carboxylation exacerbated the ear swelling responses. Analysis of the skin after MWCNT exposure reveals that the same MWCNTs with a high level of carboxylation increase epidermal thickness, mast cell and basophil degranulation, and lead to increases in polymorphonuclear cell recruitment when co-administered with 2, 4-dinitrofluorobenzene.

**Conclusions:**

The data presented here suggest that acute, topical application of low doses of MWCNTs can induce keratinocyte cytotoxicity and exacerbation of allergic skin conditions in a carboxylation dependent manner.

**Electronic supplementary material:**

The online version of this article (10.1186/s12989-018-0285-x) contains supplementary material, which is available to authorized users.

## Background

Carbon nanotubes (CNT) are layers of sp^2^ hybridized carbon atoms formed into single-walled (SWCNT) or multi-walled (MWCNT) cylindrical structures that were first reported by Sumio Iijima [1, 2]. These high aspect ratio nanoparticles have a pore diameter < 100 nm and a length usually on the micron scale. Their composition imparts carbon nanotubes with unique physical properties, including both high tensile strength and electrical conductivity. The material science field has already exploited these properties to enhance polymers used in vehicles and sports equipment; however, the expected levels of CNTs released from these products remains low [3]. Of greater concern are both the current occupational exposures from the manufacture of CNTs, and the potential exposures from future biomedical technologies that utilize CNTs.

Early assessments of CNT manufacturing and handling practices revealed that airborne CNT concentrations could be as high as 53 μg/m^3^ [4]. Research also suggested that in vivo respiratory exposures to CNTs led to acute lung inflammation and fibrosis [5–8]. These revelations prompted the National Institute for Occupational Safety and Health to recommend an 8 h time-weighted average of elemental carbon respirable mass exposure limit of 1 μg/m^3^ [9]. More recent surveys of CNT manufacturers reveal that most sites are below the new airborne exposure limit [10, 11]; however, less is known about dermal exposures to CNTs. Based on the average surface area of an adult hand (414.4 cm^2^) and the estimated deposition of CNTs during manufacturing processes of 0.2–6 mg per hand, we estimate potential dermal exposures to be anywhere from 0.5–14.5 μg/cm^2^ [4, 12]. Due to the use of personal protective equipment, it is likely that only a small fraction of the deposited CNTs contact skin; therefore, examination of low dose dermal exposure of CNTs is more relevant. Even with increased use of personal protective equipment, there is still evidence for dermal exposure of CNTs to the hands and wrists of workers [11]. Aside from occupational exposures, new biomedical technologies are being developed that could increase skin exposure to CNTs. Specifically, CNTs are being developed as transdermal drug delivery devices [13–15], bio-sensing patches [16–19], and “smart” textiles [20–22]. While these technologies are in the early stages of development, additional dermal toxicological studies of CNTs are warranted.

There are a number of studies exploring the in vitro toxicity of CNTs on skin cells, including keratinocytes and fibroblasts. Investigations into pristine SWCNT toxicity suggested that these nanoparticles induce oxidative stress and decrease the viability of an immortalized keratinocyte cell line (HaCaT) [23, 24]. A separate study found that pristine MWCNT treatment decreased cell viability and altered protein expression in human epidermal keratinocytes (HEK) [25, 26]. Pristine, unmodified CNTs are hydrophobic; therefore, they are often functionalized to enhance stability in biologic media. An examination of carboxylated MWCNTs found that they were genotoxic and decreased cell viability in normal human dermal fibroblasts [27]. A more recent study also found carboxylated MWCNTs induce DNA damage in HaCaT cells [28]. While in vitro toxicology screening suggests a potential for CNTs to induce dose dependent cell death, in vivo studies are required to identify whether this translates to skin irritation.

Comparatively, there are few in vivo dermal toxicology studies on the effects of topically applied CNTs. Two separate studies examined topical applications of high doses (> 2 mg/cm^2^) of pristine SWCNT and pristine MWCNT in rabbit and guinea pig models to assess dermal irritation. The findings suggest that only one type of pristine MWCNT led to mild erythema that resolved after 24 h [29, 30]. Another lab examined topical applications of CNTs in vivo and found that SWCNTs increased skin thickness, neutrophil activation, and mast cell recruitment in the skin of mice; however, these findings are predominantly due to the high iron content of the unpurified SWCNTs [31]. Due to the increasing prevalence of allergic skin disorders such as atopic dermatitis and allergic contact dermatitis [32, 33], it is important to examine the toxicity of CNTs in both healthy and disease models. Recently, our lab reported that a relatively low dose of 1 μg/ear of carboxylated MWCNT could significantly augment the 2,4-dinitrofluorobenzene (DNFB) induced contact hypersensitivity (CHS) response in a hairless C57BL/6 mouse model, and this response was associated with an increase in mast cell degranulation [34].

Here we examine the properties of this specific carboxylated MWCNT that led to the increase in DNFB induced CHS ear swelling. The focus of this study is on how the level of surface carboxylation on the MWCNTs can alter not only the CHS response, but also the level of in vitro keratinocyte cytotoxicity. Furthermore, a more comprehensive examination of the skin histology, immune cell infiltration, and cytokine release after topical application of the carboxylated MWCNT and DNFB elucidates possible mechanisms of action. Importantly, we have identified a threshold level of carboxylation that induces proinflammatory effects.

## Results

### MWCNT physical characterization

**Fig. 1:**
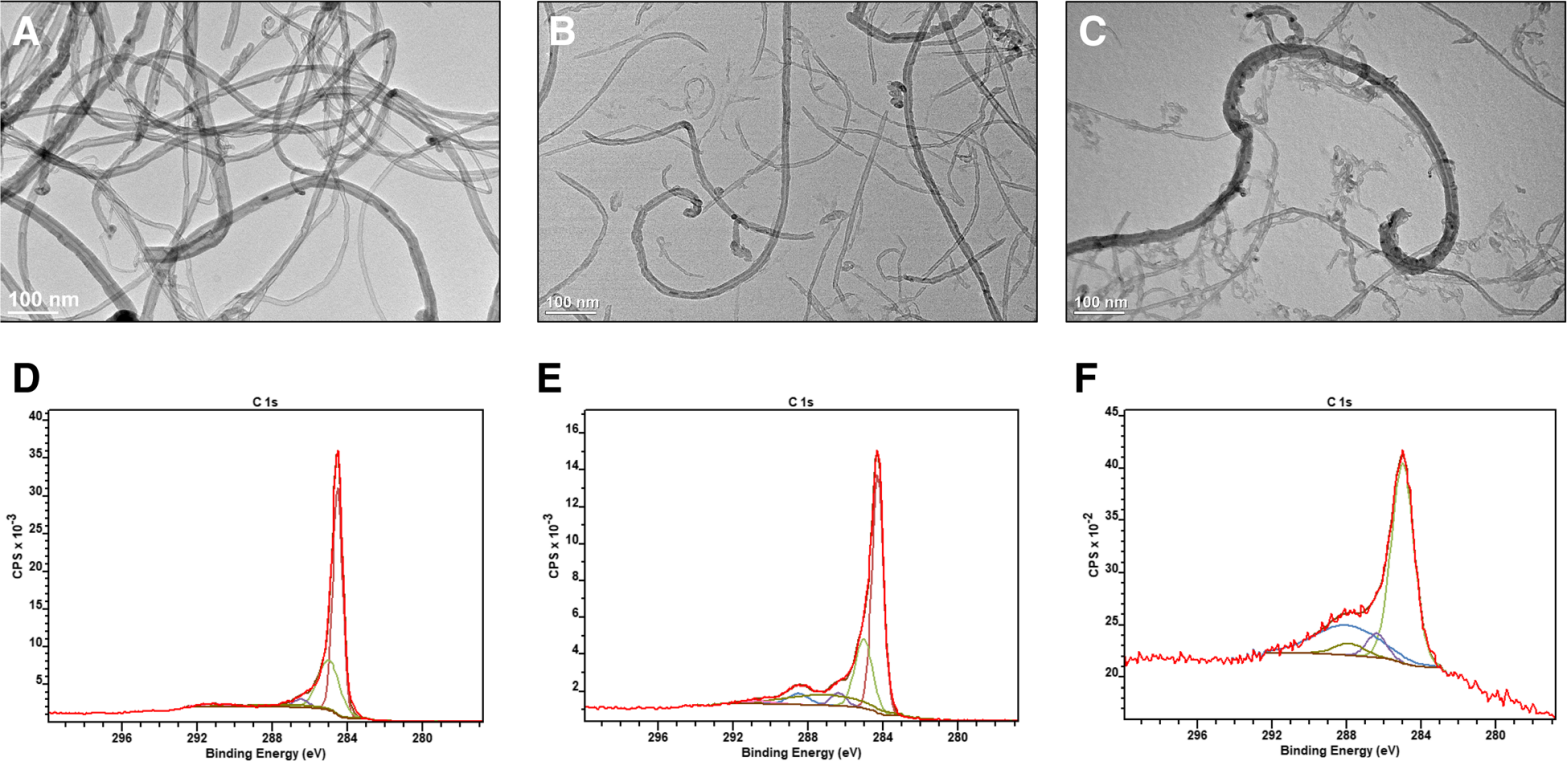
Pristine and carboxylated MWCNT TEM images and representative XPS plots. The 1 mg/mL suspensions of each nanoparticle were dried onto TEM grids for 30 s, and representative TEM images display the general structure of the pristine MWCNT (**a**), MWCNT Low-COOH (Lot #2) (**b**) and MWCNT High-COOH (Lot #1) (**c**). Representative XPS plots of the carbon 1 s regions for both the pristine MWCNT (**d**), MWCNT Low-COOH (**e**) and MWCNT High-COOH (**f**) display the relative increase in oxidized carbon bonds

The carboxylated MWCNTs previously used by our lab were reported to have a 30 nm outer pore diameter and a 5–20 μm length, suspended in water at a concentration of 1 mg/mL (Lot #1). To accompany Lot #1 in mechanistic studies, a second batch of carboxylated MWCNTs, with the same product code, were purchased from the same vendor (Lot #2). A pristine, unmodified MWCNT with the same dimensions as both carboxylated MWCNT samples will serve as a control. Both carboxylated nanoparticles were relatively well dispersed as evidenced by the low hydrodynamic diameter and polydispersity index values; however, as expected, the pristine MWCNT displayed poor dispersity in water. The dispersity in water is a direct result of the highly negative zeta potential of both carboxylated nanoparticles, which decreases particle agglomeration (Table 1). In addition, all MWCNTs were similar in overall size and shape as indicated by the representative TEM images (Fig. 1a-c) with a minor difference being the appearance of less defects and nanotube fragments in both the pristine MWCNTs and the MWCNTs of Lot #2. The presence of defects and fragments is likely a result of increased oxidation as it is well established that the oxidation process inherently induces fragments and surface defects into the CNTs [35]. To assess the level of oxidation/carboxylation, each nanoparticle was analyzed via x-ray photoelectron spectroscopy (XPS). In this technique, a high energy x-ray beam is used to eject core electrons from the sample surface. The kinetic energy of these electrons is measured to calculate the electron binding energy, which is quantitatively related to the elemental composition of the material. Analysis of the carbon 1 s electrons (Fig. 1d-f) showed unexpected differences between the different MWCNTs. First, the carbon–carbon (sp2 and sp3) bonds made up 90.83% of the pristine MWCNT, 77.69% of Lot #2, and 58.83% of Lot #1 MWCNTs. The remaining carbon atoms in each MWCNT lot were bound to oxygen in alcohol, carbonyl, or carboxylic acid functional groups. Predictably, the pristine had only minimal signs of oxidation from the acid washing process; however, the MWCNTs from Lot #1 had a two-fold higher level of carboxylation than Lot #2, which henceforth will be termed MWCNT High-COOH and MWCNT Low-COOH, respectively. Unmodified CNTs are comprised of sp^2^ hybridized carbon bonds, and surface defects lead to a higher level of sp^3^ hybridized carbon bonds. As expected, the MWCNT High-COOH nanoparticles have a much lower sp^2^/sp^3^ ratio compared to either the pristine or Low-COOH MWCNTs (Table 1). MWCNTs are often made with transition metal catalysts, which can induce toxicity as well [31]. These nanoparticles were acid washed and carboxylated which removed any trace of transition metals as detected by the XPS elemental survey (Additional file 1: Table S1).

**Table 1:** Carboxylated MWCNT physical characterization

MWCNT	Hydrodynamic Diameter (nm)	Polydispersity Index	Zeta Potential (mV)	C-C (sp^2^/sp^3^)	C-OH (%)	C=O (%)	HO-C=O (%)
High-COOH (Lot #1)	284.3	0.459	−42.1	0.26	13.88	7.59	19.7
Low-COOH (Lot #2)	178.5	0.268	−54.0	1.47	4.16	8.73	9.42
Pristine	> LOD^a^	0.865	6.79	2.06	2.66	4.66	0

The nanoparticle suspensions of pristine, Lot #1, and Lot #2 MWCNTs were diluted in water and analyzed in a Malvern zetasizer. The hydrodynamic diameter, polydispersity index, and zeta potential are presented in the table. Also, suspensions of each nanoparticle were dried onto silicon wafers and analyzed via XPS, and information on the carbon region are presented in the table. The sp^2^/sp^3^ carbon bond ratio is the percentage of sp^2^ carbon bonds divided by the sp^3^ carbon bonds on the surface and indicates the level of surface defects. The C-OH (alcohol), C=O (carbonyl), and HO-C=O (carboxyl) functional groups are all represented as a percent of total surface carbon bonds. ^a^ The hydrodynamic diameter for the pristine MWCNTs was above the machines limit of detection

### MWCNT oxidation dependent exacerbation of DNFB induced CHS

**Fig. 2:**
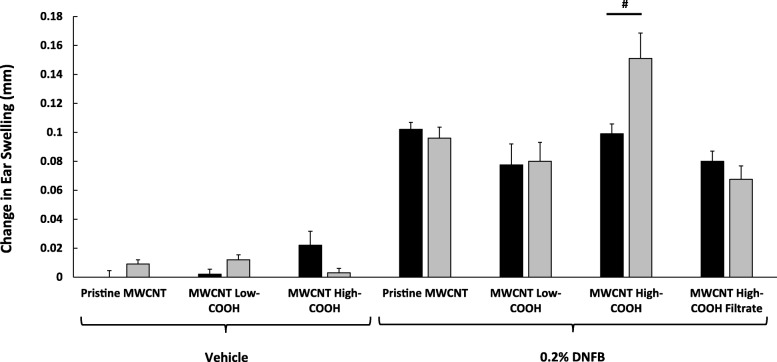
Effects of MWCNT on the DNFB induced CHS ear swelling responses after 24 h. Mice were sensitized with 30 μl of 0.05% DNFB in a 4:1 acetone/olive oil vehicle on the lower dorsum on day 0. On day 5, the mice were challenged with either vehicle or 0.2% DNFB. The right ears (black bars) are controls that were not treated with MWCNT. The left ears (gray bars) were treated with either pristine MWCNT, MWCNT Low-COOH, MWCNT High-COOH, or MWCNT High-COOH 0.45 μm filtrate. The DNFB increased ear swelling significantly, compared to all vehicle treated mice. Only ears treated with DNFB and MWCNT High-COOH displayed significantly increased swelling responses, compared to DNFB only. Graphs represent changes in mean ear swelling after 24 h (+/− SEM), *N* = 5. A Student’s T-test was used to analyze all data presented here, since comparisons were between corresponding right and left ears. Significance is defined at a *p*-value < 0.05, and # indicates significance between groups

Interestingly, we have shown that the MWCNT High-COOH particles induce greater keratinocyte cell death in vitro, when compared to either pristine MWCNT or MWCNT Low-COOH particles (Additional file 1: Figure S1A). However, pristine MWCNT produce far more reactive oxygen species (ROS) than either oxidized MWCNT treatment, in cultured keratinocytes, indicating that the oxidized MWCNT induced cell death is largely ROS independent (Additional file 1: Figure S1B). The in vitro data is suggestive of potential MWCNT induced dermal irritation, and to examine the effect of these MWCNTs on normal and inflamed skin, our lab uses hairless C57BL/6 mice treated with either vehicle or a DNFB sensitizer. As we have previously reported, topically applied MWCNT High-COOH particles exacerbate the DNFB induced CHS ear swelling response 24 h after co-challenge with 0.2% DNFB and 1 μg/ear MWCNT High-COOH [34]. This response appears to be synergistic as the MWCNT High-COOH particles have no effect on ear swelling without the sensitizer (Fig. 2). The MWCNT High-COOH particles have a high level of surface defects and particle fragments as indicated by TEM. To assess whether these small fragments are responsible for the increased swelling response, the MWCNT High-COOH particles were filtered through a 0.45 μm filter and the filtrate was used to co-challenge with 0.2% DNFB. The 0.45 μm filtrate was unable to augment the ear swelling response; therefore, the larger MWCNTs are likely the source of increase ear swelling in the CHS model (Fig. 2). Lastly, the MWCNT Low-COOH and pristine MWCNT particles were also tested at a concentration of 1 μg/ear; however, these particles had no effect on ear swelling, when co-treated with either vehicle or 0.2% DNFB (Fig. 2). The data suggests that only MWCNT with a high level of surface carboxylation will exacerbate DNFB induced CHS ear swelling.

### Characterization of MWCNT High-COOH Induced Skin Inflammation in a CHS Model

Only the MWCNT High-COOH particles exacerbated DNFB induced CHS ear swelling, and this nanoparticle also reduced the cell viability of cultured keratinocytes more than its pristine or Low-COOH counterpart. To further assess the possible in vivo mechanisms of action of this augmented allergic skin response, a 5 cm^2^ area of mouse dorsal skin was challenged with either vehicle, 1 μg/cm^2^ of MWCNT High-COOH particles alone, 0.2% DNFB alone, or MWCNT High-COOH at 1 μg/cm^2^ plus 0.2% DNFB. After 24 h, the mice were euthanized and the skin histology, immune cell infiltrates, and cytokine expression were examined.

**Fig. 3:**
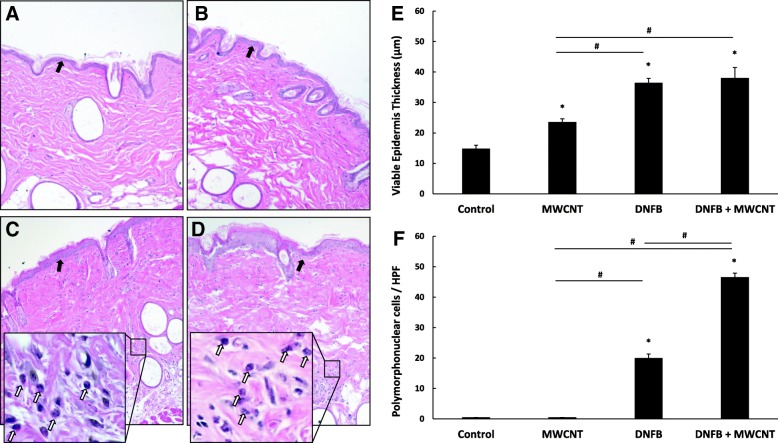
MWCNT High-COOH nanoparticles induce increases in epidermal thickness and PMN recruitment in skin. Mice were sensitized with 30 μl 0.05% DNFB in a 4:1 acetone/olive oil vehicle on the abdomen on day 0. On day 5 the mice were collared to prevent grooming and challenged with 50 μl of either vehicle (**a**), 5 μg MWCNT (**b**), 0.2% DNFB (**c**), or 0.2% DNFB with 5 μg MWCNT (**d**) on a 5 cm^2^ area of dorsal skin for 24 h. Skin was fixed in 10% formalin, paraffin embedded, and stained with hematoxylin and eosin to assess the skin histology. Representative images of H&E stained samples are included, and the black arrows indicate the epidermis in each image. Higher magnification images of representative polymorphonuclear cells (PMN) are included, and white arrows indicate the PMN cells. The epidermal skin thickness (**e**) and total number of PMN cells (**f**) are quantified. The graphs represent the mean (+/− SEM), *N* = 6. Significance is defined at a p-value < 0.05. * indicates significance compared to control, # indicate significance within groups

First, histology was examined on H&E stained skin and the average viable epidermal thickness was measured. It is noteworthy that even the MWCNT High-COOH treated skin had a significantly thicker epidermis, compared to control skin. Skin irritants are known to elicit epidermal hyperproliferation and increased skin thickness [36]. DNFB is both a sensitizer and a skin irritant [37], and as expected, DNFB caused the greatest increase in epidermal thickness in both the DNFB group and the co-treatment of both DNFB and the MWCNT High-COOH (Fig. 3e). It is also noteworthy, that both DNFB treated skin sections displayed increased cellular infiltration in the dermis. A large portion of these cellular infiltrates were polymorphonuclear cells (PMN), and the DNFB and MWCNT High-COOH co-treatment had significantly more PMNs compared to DNFB only treated skin (Fig. 3f).

**Fig. 4:**
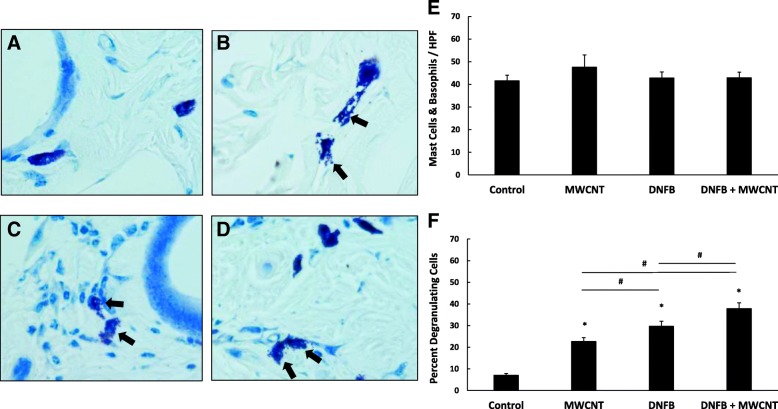
MWCNT High-COOH nanoparticles induce mast cell/ basophil degranulation in skin. Mice were sensitized with 30ul 0.05% DNFB in a 4:1 acetone/olive oil vehicle on the abdomen on day 0. On day 5 the mice were collared to prevent grooming and challenged with 50 μl of either vehicle (**a**), 5 μg MWCNT (**b**), 0.2% DNFB (**c**), or 0.2% DNFB with 5 μg MWCNT (**d**) on a 5 cm^2^ area of dorsal skin for 24 h. Skin was fixed in 10% formalin, paraffin embedded, and stained with toluidine blue to assess the number of degranulating mast cells/basophils. Representative images of toluidine blue stained samples are included, and black arrows indicate degranulating mast cells. The total number of mast cells/ basophils (**e**) and the percentage of degranulating mast cells/ basophils (**f**) are quantified. The graphs represent the mean (+/− SEM), N = 6. Significance is defined at a p-value < 0.05. * indicates significance compared to control, # indicate significance within groups

We previously reported that the exacerbation of CHS induced ear swelling caused by the MWCNT High-COOH was associated with increased mast cell degranulation [34]. Here we examined the levels of both mast cell and basophil degranulation by counting the number of degranulating and intact cells in toluidine blue stained skin sections. The total number of mast cell or basophils per field was unchanged (Fig. 4e); however, the percentage of degranulating mast cells or basophils was significantly increased by MWCNT High-COOH treatment (Fig. 4f). DNFB also increased the percent of degranulating cells, when compared to control. The MWCNT and DNFB co-treatment significantly increased degranulation when compared to DNFB treatment only. Interestingly, the increased percent of degranulation observed after the MWCNT treatment does not translate to an increase in skin swelling as shown in Fig. 2, which indicates that mast cell or basophil degranulation alone was not sufficient to induce skin swelling in this model.

**Fig. 5:**
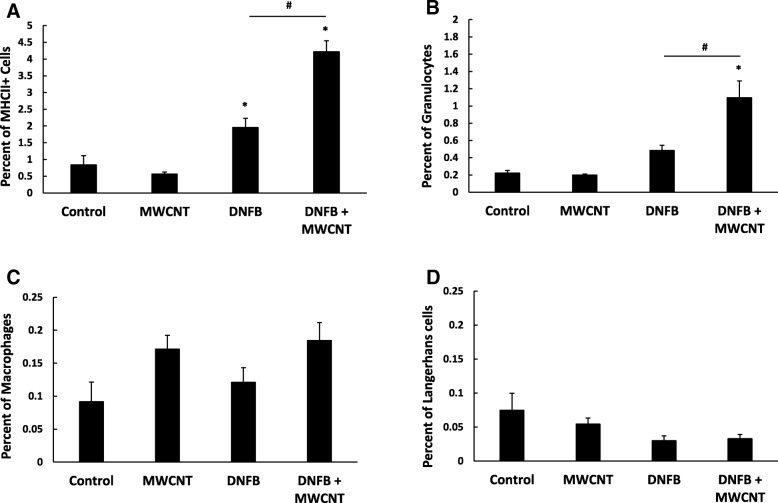
MWCNT High-COOH nanoparticles exacerbate DNFB induced MHCII skin cell expression and granulocyte infiltration. Mice were sensitized with 30 μl 0.05% DNFB in a 4:1 acetone/olive oil vehicle on the abdomen on day 0. On day 5 the mice were collared to prevent grooming and challenged with 50 μl of either vehicle, 5 μg MWCNT, 0.2% DNFB, or 0.2% DNFB with 5 μg MWCNT on a 5 cm^2^ area of dorsal skin for 24 h. A 2 cm^2^ section of skin was processed into a single cell suspension and stained for flow cytometry. The cell populations were gated into total APCs (MHCII+) (**a**), granulocytes (CD11b+, F4/80-, SSC high) (**b**), macrophages (MHCII+, F4/80+, CD11c-) (**c**), and Langerhans cells (MHCII+, F4/80-, CD11c+) (**d**). The graphs represent the mean percentage of positive cells in the total live cell population (+/− SEM), N = 6. Significance is defined at a p-value < 0.05. * indicates significance compared to control, # indicate significance within groups

In the H&E stained skin, immune cell infiltration was observed in the dermis of both the DNFB treated samples (Fig. 3f). To more fully characterize the cellular infiltrates, the skin was analyzed by flow cytometry. The total percentage of antigen presenting cells (APCs) was determined by major histocompatibility complex II (MHCII) staining. There is a significantly higher percentage of MHCII stained cells in both DNFB treated samples when compared to control or MWCNT High-COOH treated skin, but the DNFB and MWCNT High-COOH co-treatment induced the highest percentage of skin APCs (Fig. 5a). The percentage of granulocytes (CD11b+, F4/80-, and high side scatter) in the skin displayed a slight, nonsignificant increase due to DNFB alone; however, significantly more granulocytes were present in skin co-treated with both DNFB and MWCNT High-COOH (Fig. 5b). These granulocytes are most likely neutrophils, since toluidine blue histology suggested that the total number of mast cells and basophils remained unchanged, and DNFB is a T_H_1 skewing sensitizer that is unlikely to recruit high levels of eosinophils [38, 39]. While the percentage of macrophages (MHCII+, F4/80+, CD11c-) appeared to increase due to MWCNT High-COOH treatment, the increases are nonsignificant (Fig. 5c). Lastly, the percentage of Langerhans cells (MHCII+, F4/80+, CD11c+) in the skin was relatively low, and was slightly reduced due to DNFB treatment (Fig. 5d) which is consistent with their trafficking out of skin to the local lymph node. It should be noted, that the total percentage of granulocytes, macrophages, or Langerhans cells did not equal the total MHCII positive skin cell percentage. The remaining cells are most likely B cells or keratinocytes, as keratinocytes are reported to express MHCII under inflammatory conditions [40].

**Fig. 6:**
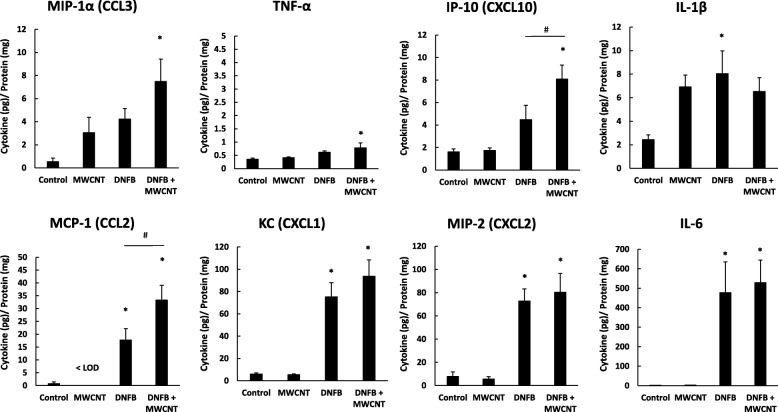
MWCNT High-COOH nanoparticles augment skin expression of inflammatory cytokines. Mice were sensitized with 30 μl 0.05% DNFB in a 4:1 acetone/olive oil vehicle on the abdomen on day 0. On day 5 the mice were collared to prevent grooming and challenged with 50 μl of either vehicle, 5 μg MWCNT, 0.2% DNFB, or 0.2% DNFB with 5 μg MWCNT on a 5 cm^2^ area of dorsal skin for 24 h. A 1 cm^2^ piece of skin was homogenized and the cytokine protein expression levels were analyzed by Luminex assay. The graphs represent the mean (+/− SEM), N = 6. All data was analyzed by two-way ANOVA except for MCP-1, which was analyzed by one-way ANOVA, due to some data being below the limit of detection. Significance is defined at a p-value < 0.05. * indicates significance compared to control, # indicate significance within groups

To determine signaling events that prompted the difference in immune cell infiltration in the skin, we analyzed cytokine and chemokine protein expression in the skin 24 h after treatment. DNFB is highly inflammatory and led to significant increases in most cytokines analyzed. Only DNFB had significant effects on Interleukin 6 (IL-6), keratinocyte chemoattractant (KC), macrophage inflammatory protein 2 (MIP-2), and tumor necrosis factor alpha (TNFα) (Fig. 6). IL-6 and TNFα are both inflammatory cytokines produced by a number of cells in the skin, including keratinocytes [41]. KC and MIP-2 are both neutrophil chemotactic cytokines produced by macrophages and keratinocytes [42, 43]. DNFB and MWCNT High-COOH co-treatment significantly enhanced the expression of monocyte chemoattractant protein 1 (MCP-1) and interferon gamma inducible protein 10 (IP-10); however, this is a synergistic effect, since MWCNT High-COOH treatment alone had no effect on these cytokines. MCP-1 is produced by monocytes, macrophages, or dendritic cells and can induce degranulation of mast cells or basophils [44]. IP-10 is produced in response to IFNγ, and is chemotactic for T cells [45]. It is noteworthy that skin treated with MWCNT High-COOH alone trended toward an increase of both macrophage inflammatory protein 1 alpha (MIP-1α) and interleukin 1 beta (IL-1β) by 2–6 fold, compared to control. MIP-1α is predominantly produced by macrophages, and it is involved in the activation of neutrophils [46]. IL-1β is part of the inflammasome, and it is released from a number of cells, including keratinocytes [41]. Overall, the MWCNT treatment affected cytokines involved in both neutrophil and T cell recruitment.

### Determining a Threshold of MWCNT Carboxylation for Induction of Skin Toxicity

To confirm that the observations above correlate with the level of carboxylation and to determine the level of carboxylation that induces skin toxicity, the pristine MWCNTs characterized in Fig. 1 were acid oxidized in-house for either 1.5, 3, 6, or 12 h. The pristine MWCNTs are neutrally charged and highly agglomerated, while the oxidized MWCNTs are all negatively charged and well dispersed in water (Table 2). All oxidized MWCNTs, except for those oxidized for 1.5 h, have a sp^2^/sp^3^ ratio below 1, suggesting a high degree of surface defects. All oxidized MWCNTs have detectable carboxylic acid groups, and the 1.5, 3, 6, and 12 h oxidized MWCNTs have 9.66, 12.96, 30.61, and 26.7% carboxylated surface carbons, respectively (Table 2, Additional file 1: Figure S2). It appears as though 25–30% surface carboxylation is the limit achieved by this acid oxidation, and TEM images indicate that by 12 h significant nanotube fragmentation began to occur (Additional file 1: Figure S3).

**Table 2:** In-house Oxidized MWCNT physical characterization

MWCNT	Hydrodynamic Diameter (nm)	Polydispersity Index	Zeta Potential (mV)	C-C (sp^2^/sp^3^)	C-OH (%)	C=O (%)	HO-C=O (%)
1.5 Hour Oxidation	373.0	0.461	−66.3	1.54	28.78	3.79	9.66
3 Hour Oxidation	332.3	0.459	−68.8	0.79	26.91	3.95	12.96
6 Hour Oxidation	208.8	0.363	−48.7	0.57	6.09	0.2	30.61
12 Hour Oxidation	178.9	0.354	−61.9	0.72	5.33	0.01	26.7

The nanoparticle suspensions of 1.5 h oxidized, 3 h oxidized, 6 h oxidized, and 12 h oxidized MWCNTs were diluted in water and analyzed in a Malvern zetasizer. The hydrodynamic diameter, polydispersity index, and zeta potential are presented in the table. Also, suspensions of each nanoparticle were dried onto silicon wafers and analyzed via XPS, and information on the carbon region are presented in the table. The sp^2^/sp^3^ carbon bond ratio is the percentage of sp^2^ carbon bonds divided by the sp^3^ carbon bonds on the surface and indicates the level of surface defects. The C-OH (alcohol), C=O (carbonyl), and HO-C=O (carboxyl) functional groups are all represented as a percent of total surface carbon bonds

**Fig. 7:**
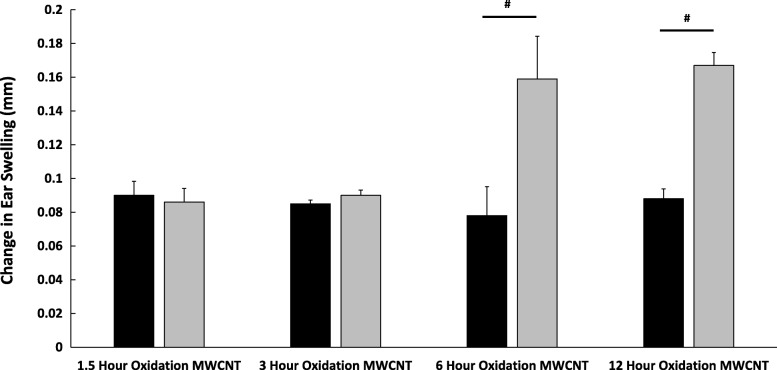
MWCNTs oxidized for 6 or more hours exacerbate the CHS ear swelling response. Mice were sensitized with 30 μl 0.05% DNFB in a 4:1 acetone/olive oil vehicle on the lower dorsum on day 0. On day 5 the mice were challenged with 20 μl per ear of 0.2% DNFB on the right ear (black bars) and 0.2% DNFB with 1 μg/ear of the different oxidized MWCNTs on the left ear (gray bars). Graphs represent the change in mean ear swelling, after 24 hours, (+/- SEM), N=5. Data were analyzed by a Student’s T-test, comparing the DNFB and DNFB + MWCNT groups. Significance is defined at a *p*-value < 0.05. # indicate significance compared to DNFB control

To examine the effect of carboxylation level in vivo we co-treated the ears of sensitized mice with DNFB on the right ear (black bars) and DNFB with 1 μg/ear of the oxidized MWCNTs on the left ear (gray bars) (Fig. 7). Both MWCNT oxidized for 1.5 and 3 h had no effect on the DNFB induced ear swelling; however, both the 6 and 12 h oxidized MWCNT significantly increased in the ear swelling response, compared to ears treated with only DNFB (Fig. 7). These data confirm that MWCNT with > 20% surface carboxylation exacerbate DNFB induced CHS responses compared to less oxidized MWCNT.

## Discussion

Acute exposures to low doses of highly oxidized MWCNTs lead to epidermal irritation characterized by increased keratinocyte proliferation, cytokine release, and mast cell or basophil degranulation. While the MWCNTs alone are not sufficient to induce skin swelling, they may exacerbate allergen related skin inflammation, primarily through the enhanced recruitment of neutrophils. This trend of carboxylation dependent effects was also observed in cell culture, as HaCaT keratinocytes displayed increased cytotoxicity when exposed to MWCNTs with high levels of carboxylation; however, the increased cytotoxicity was unrelated to ROS generation. To our knowledge, our lab is the first to report this carboxylation dependent MWCNT dermal toxicity, in vivo.

CHS is a biphasic, delayed-type immunity that requires interactions and signaling from a number of different immune cells. First, the sensitization phase produces memory T cells that recognize a specific antigen [47]. In the subsequent challenge phase, the allergen promotes early, innate immune events that depend on keratinocyte inflammatory cytokine release [48], mast cell activation [49], and neutrophil recruitment [50]. Following these early events, antigen specific cytotoxic CD8+ and effector CD4+ T cells infiltrate skin and lead to skin swelling [51]. Due to the large size of the MWCNTs, it is unlikely the nanoparticles directly interact with cells in the dermis (e.g. mast cells or T cells). Therefore, we focused on the early innate immune responses of CHS, mediated by keratinocytes and APCs at the skin surface.

The mechanism of MWCNT exacerbation of DNFB induced CHS appears to be mediated through epidermal irritation. Both DNFB and MWCNT increased the epidermal thickness in the mouse skin, which is often a response to barrier disruption. The in vitro data displaying increased HaCaT cytotoxicity with increasing surface carboxylation level is also suggestive of epidermal irritation. It is plausible that either the carboxylic acid groups have a special reactivity with epidermal keratinocytes, or the oxidation enhances the dispersity of the tubes to allow for enhanced interaction with viable epidermal cells. It is noteworthy that the MWCNT oxidized for 3 h display more total oxidation than the MWCNT oxidized for 6 or 12 h, but this oxidation is limited to mostly alcohol functional groups. Compared to the other oxidized MWCNTs, the MWCNT oxidized for 3 h have approximately half the amount of carboxylic acid surface functionalization, and these nanoparticles do not affect the in vivo CHS responses. In the future, the effect of surface functionalization density on MWCNT toxicity should be examined for other functional groups as well.

This study also identified mast cell or basophil degranulation as a key event in the MWCNT exacerbation of DNFB induced CHS. The MWCNT treatment increased degranulation in both the control and DNFB treated mice. While we were unable to identify a direct signal that led to mast cell and basophil degranulation, it is likely that the inflammatory skin environment produced by MWCNT induced epidermal irritation is the primary cause. MCP-1 is known to induce mast cell degranulation in some tissues [44], and MCP-1 was elevated by MWCNT in the DNFB treated mice. However, MCP-1 was not detected in animals exposed to only MWCNT; therefore, MCP-1 expression alone does not explain the MWCNT induced mast cell and basophil degranulation. MIP-1 α expression was upregulated in skin due to MWCNT exposure, and this cytokine has been described as a co-stimulatory signal for mast cell degranulation in the conjunctiva, meaning it is required for degranulation, but not sufficient to induce degranulation alone [52]. It is possible that the primary degranulation signal is complement activation and signaling through IgM [53]; however, this mechanism would require specific antigen recognition. Alternatively, skin irritation, which results in keratinocyte necrosis, could release damage associated molecular patterns. Some of these proteins have been identified to induce mast cell degranulation [54]. Mast cell degranulation and activation leads to the release of inflammatory cytokines, histamine, prostaglandins, and leukotrienes. These mediators cause vasodilation and enhance immune cell extravasation through endothelial cells [55], which has implications for immune cell infiltration in CHS responses.

There is evidence that suggests mast cell activation is a prerequisite for neutrophil infiltration into tissues [56, 57]. In this study, we identified an increase in granulocyte skin infiltration after DNFB exposure, and MWCNT exacerbated this DNFB induced effect. However, mast cell degranulation is not sufficient to induced neutrophil recruitment alone, since granulocyte infiltration was unchanged by MWCNT treatment in control skin. DNFB exposure led to increased KC and MIP-2 cytokine expression, both of which are potent neutrophil chemotactic cytokines necessary for CHS [42, 43]. More recently, MIP-1α and MCP-1 have been identified as factors that recruit and activate neutrophils by enhancing extravasation and increasing the expression of lipid mediators [46]. These two cytokines were upregulated by MWCNT skin exposure, and along with mast cell degranulation could explain the increased granulocyte infiltration in skin. Neutrophils enhance skin inflammation through cytokine and protease release; however [58, 59], they also have a role in T cell recruitment to enhance CHS responses. Neutrophils, along with other APCs, are known to express IP-10, a chemotactic cytokine for the recruitment of T cells [45], and IP-10 expression follows a similar pattern as neutrophil infiltration in our model. Studies suggest that level of neutrophil infiltration is correlated with the level of cytotoxic T cell infiltration in the later phases of CHS [60]. We conclude that both direct effects of enhanced neutrophil infiltration, and indirect effects of subsequent cytotoxic T cell recruitment play a role in the MWCNT exacerbation of DNFB induced CHS skin swelling.

The use of mouse models for dermal nanotoxicology are useful since they offer insight into immune cell activation that keratinocyte cell culture alone cannot provide. However, mouse skin differs from human skin in both thickness and hair follicle density [61]. The thickness of the epidermis in humans can be anywhere from 2-15x thicker than the mouse, depending on the area. Therefore, mouse skin exposures to nanoparticles are more representative of worst-case exposures to barrier disrupted human skin. Highly oxidized MWCNT should be examined in reconstructed human epidermal cultures to identify if they induce the same level of skin irritation. However, the data presented here may be relevant for a large population of individuals with skin inflammatory conditions [32, 33]. People with inflammatory skin conditions have barrier disrupted skin that makes nanoparticle exposure to the viable epidermis more likely [62].

The finding that highly carboxylated MWCNT exacerbate allergic skin responses is in line with data published suggesting that MWCNT exposure to the lung exacerbates allergic airway inflammation. Early reports indicate that inhaled or instilled MWCNT exacerbate ovalbumin induced airway inflammation [63, 64]. More recently, the effects of MWCNT on allergic airway inflammation seem to be caused by inflammasome activation and innate cytokine production, like IL-33 [65–67]. However, these reports on MWCNT exacerbating allergic airway inflammation examined pristine, unmodified MWCNT. Studies on the lung inflammation of oxidized MWCNT suggest that functionalization decreases bioactivity, compared to unmodified MWCNT [68, 69]. While the level of surface carboxylation was not mentioned in either study, it is likely that oxidized MWCNT toxicity is both exposure route and tissue specific.

While the in vitro cytotoxicity data and in vivo CHS ear swelling data presented here are well correlated, the in vitro ROS data displays an opposite trend. The ROS data would suggest that the pristine tubes would exert a high level of ROS dependent toxicity in skin; however these nanoparticles had no effect on the CHS response. It is important to remember the differences in dosimetry between in vitro and in vivo nanoparticle exposures that can make interpretation of in vitro data difficult. This could explain why the in vitro data on the toxicity of functionalized MWCNT in the literature displays less consensus. Early studies suggested that increasing the surface functionalization density of SWCNT decreased the cytotoxicity in an in vitro human dermal fibroblast model [70]. Examination of carboxylated MWCNT in a THP-1 monocyte cell line found that the functionalization decreased inflammasome activation [71]. More recent examination of carboxylated MWCNT in a keratinocyte cell line found that the MWCNT induced genotoxic DNA damage, regardless of functionalization [28]. Lastly, there are reports of MWCNT induced macrophage cell line cytotoxicity increasing with higher levels of oxidation [72]. The lack of consensus in these reports is likely due to differences in particle characteristics, doses, and cell lines. Altogether, this is evidence of the need for more consistent in vitro toxicity models and increased nanoparticle physical characterization.

## Conclusions

This study has identified a MWCNT carboxylation dependent keratinocyte toxicity that is not mediated by ROS generation. Low, physiologically relevant exposures to the highly oxidized MWCNT also lead to epidermal irritation, cytokine release, and mast cell degranulation in a mouse model. These MWCNT skin effects exacerbate DNFB induced CHS reactions by increasing neutrophil skin recruitment. Altogether, this data suggests that more dermal toxicity studies of oxidized MWCNT exposures should be conducted before widespread commercial use. Alternatively, more biocompatible alternative functional groups should be explored, to reduce toxicity.

## Methods

### Carbon Nanotube Oxidation and Characterization

The MWCNT (High COOH) and MWCNT (Low COOH) were two different nanoparticle lots purchased from Nanolab (Cat# PD30L5–20COOH) and they were suspended in water at a 1 mg/mL concentration. The MWCNT (Pristine) particles were also purchased from Nanolab (Cat# PD30L5–20), but these were in powder form. All of the carbon nanotubes used in this study have a pore diameter of 30 nm and a length ranging from 5 to 20 μm.

To oxidize the carbon nanotubes, 100 mg of dry MWCNT (Pristine) were placed into a round bottom flask containing 100 mL of a 4:1 mixture of concentrated sulfuric acid and 70% nitric acid. The mixture was refluxed, while stirring at 80^o^ C for 3, 6, or 12 h. To stop the oxidation process, the acid mixture was diluted with water, and the carbon nanotubes were centrifuged at 3000 x g for 20 min. The carbon nanotubes were washed with water two additional times. Finally, the tubes were suspended in 50 mL of water, sonicated for 15 min with a 300 W probe sonicator at a 20% duty cycle with 40% power, and neutralized with dilute sodium hydroxide. For the 0 h sample, 100 μg of dry MWCNT (Pristine) was simply placed in 50 mL of water and sonicated. Since the oxidation process results in the loss of some carbon material, the sample concentrations were confirmed by drying a known volume of the samples in a 50^o^ C oven overnight in pre-weighed crucibles.

To assess the relative size, dispersity, and charge of the MWCNT; the nanoparticles were diluted 200x in water and analyzed in the Malvern Zetasizer Nano ZS instrument to determine the hydrodynamic diameter and surface charge. The shape and structure of the MWCNT was analyzed via transmission electron microscopy (TEM). Lastly, the chemical composition of the MWCNT surface was assessed with x-ray photoelectron spectroscopy (XPS, Kratos Axis Ultra DLD). For XPS analysis, the samples were dried onto a 1 cm × 1 cm silicon wafer (p-type, 0.001 ohm-cm) in a 50^o^ C oven overnight. The complete elemental survey was completed with a 160 eV pass energy and represents the average of 5 sweeps, and the peaks were assigned by an automated elemental electron binding energy library on CasaXPS version 2.3.18 (Casa Software, Ltd., Teignmouth, UK). The dried MWCNT film was sufficiently thick that silicon was not detected in the survey scan. The carbon 1 s region was analyzed with a 20 eV pass energy and represents the average of 5 sweeps. The relative percentages of the different carbon bonds were estimated by an automated Gaussian/Laurentzian curve fitting after setting the following carbon electron binding energy peaks base on literature values: C-C sp^2^ bond (284.3–284.6 eV), C-C sp^3^ bond (285–286 eV), C-OH alcohol (286.4–286.7 eV), C=O carbonyl (287.1–288.1), HO-C=O carboxylic acid (288–289.4 eV), or pi-stacking electrons (290.5–291.5 eV) [73].

### Cell culture

The HaCaT cells used in this study are a spontaneously transformed, immortal keratinocyte cell line [74]. These adherent cells were grown in Dulbecco’s Modified Eagle Medium (DMEM) (Gibco Cat# 11965–092) supplemented with 10% fetal bovine serum (Gibco Cat# 10082–147) and 1% penicillin/streptomycin (Gibco Cat# 15140–122). The cells were incubated in 5% carbon dioxide at 37^o^ C. Before nanoparticle treatment, the cells were trypsinized with 0.25% Trypsin-EDTA (Gibco Cat# 25200–056) and seeded into two 96 well plates at a density of 35,000 cells per well. The HaCaT cells were grown for 24 h, or until they were 70% confluent.

For the reactive oxygen species (ROS) assay, the cells were cultured in a black walled 96 well plate, washed with phosphate buffered saline (PBS), and incubated with 100 μL of 10 μM DCF-DA dye (Invitrogen Cat. D399) dissolved in PBS for 30 min prior to MWCNT treatment. After loading the cells with DCF-DA, the PBS was removed, and the wells were treated with 100 μL of culture media containing 0–25 μg/mL of the MWCNT (High COOH), MWCNT (Low COOH), MWCNT (Pristine), MWCNT (3 Hour Oxidation), MWCNT (6 Hour Oxidation), or MWCNT (12 Hour Oxidation). For a positive control, 100 μL of culture media containing 100 μM of hydrogen peroxide was added to induce ROS in the HaCaTs. To measure the DCF fluorescence after 24 h, the 96 well plate fluorescence was measured in a multimode plate reader (Perkin Elmer) at an excitation wavelength of 490 nm and an emission wavelength of 525 nm. The data were then normalized to the control.

For the cell viability assay, the cells cultured in the white walled 96 well plate were directly treated with 100 μL of culture media containing 0–25 μg/mL of the MWCNT (High COOH), MWCNT (Low COOH), MWCNT (Pristine), MWCNT (3 Hour Oxidation), MWCNT (6 Hour Oxidation), or MWCNT (12 Hour Oxidation). After 24 h, 100 μL of CellTiter-Glo (Promega Cat# G7571) reagent was added to each well, without washing. After 15 min, the luminescence of each well was measured with a Turner Biosystems Modulus microplate reader. The data were then normalized to the control, which represent the relative number of metabolically active cells remaining in culture by measuring the ATP content of each well.

### Mouse contact hypersensitivity model

A hairless C57BL/6 mouse model was used for all in vivo studies. This mouse is fully immunocompetent, but possesses a gene mutation that causes alopecia after the first follicular maturation, post birth. This model is preferable, since topical treatments would necessitate hair removal in other mouse models, and shaving or depilatory agents can cause unwanted skin barrier defects. The mice examined were all male and between 5 and 6 months old, since we have identified age, but not sex-based differences in the DNFB induced CHS response of these mice [34]. The mice were housed individually in standard cages to prevent interactions that would damage the skin or lead to cross contamination of topical agents. The mice were kept on a 12 h light/dark cycle and had access to food and water ad libitum.

To measure the level of skin swelling in the contact hypersensitivity response, mice were sensitized to 30 μL 0.05% DNFB in a 4:1 acetone (Sigma Aldrich Cat# AGCN20-25 M) and olive oil (Wegman’s Brand Pure Olive Oil) mixture on the lower dorsum on day 0. This sensitization dose is lower than most doses reported in the literature; however, we have previously reported that the mouse model used in this study is more sensitive to DNFB induced skin irritation [34]. On day 5, the ear thickness of each mouse was measured with digital calipers before treatment with 20 μL 0.2% DNFB in a 4:1 acetone and olive oil mixture on the right ear (10 μL on each side of the ear). The left ear was treated with the same concentration of DNFB, but the MWCNT treatments were also added to this ear at a concentration of 1 μg/ear. On day 6, the ear thickness was measured again by digital calipers, and the difference between the day 5 and day 6 measures represents the increase in ear swelling induced by the allergen and nanoparticles.

To determine the inflammatory effect of the DNFB and the MWCNT (High COOH), the CHS protocol was altered to challenge the lower dorsum. Briefly, the abdomen of each mouse was sensitized with 30 μL 0.05% DNFB in a 4:1 acetone and olive oil mixture on day 0. On day 5, the mice were placed in Elizabethan collars to prevent grooming, and the mice were pre-treated with 50 μL of a 10:1 acetone and water mixture with or without 5 μg of MWCNT (High COOH) over a 5 cm^2^ area of the lower dorsum. After 15 min, the mice were challenged with either 50 μL of vehicle or 50 μL 0.2% DNFB in a 4:1 acetone and olive oil mixture over the same 5 cm^2^ area of the lower dorsum. On day 6, the mice were euthanized via carbon dioxide asphyxiation, and the skin was harvested for analysis.

### Histology

After euthanasia, the treated dorsal skin was immediately cut into 1 cm × 1 cm squares and fixed in 10% formalin (Electron Microscopy Sciences Cat# 15740–04) for 48 h. The fixed skin was then embedded in paraffin blocks and 5 μm transverse sections were cut with a microtome and placed on glass slides. Two sets of slides were stained with either hematoxylin/eosin (Electron Microscopy Sciences Cat# 26030–20/26051–21) for general histological examination, or 0.1% toluidine blue (MP biomedicals Cat# 152649) for examination of mast cells and basophils. The stained images were obtained using a Nikon Eclipse E800 bright field microscope. The epidermal thickness was measured using the free source ImageJ software [75]. The mast cell and basophil counts were performed by counting both the total intact and degranulating cells over ten 20x fields of view. The PMN counts were performed by counting the number of cells over ten 40x fields of view.

### Flow cytometry

Following euthanasia, 2 cm^2^ of treated dorsal skin was excised and placed into 1 mL of 1% fetal bovine serum in Hank’s Balances Salt Solution (HBSS). An additional 1 mL of 0.1 U Collagenase/ 0.8 U Dispase (Sigma Aldrich Cat# 11097113001) was added to the skin samples along with 0.3 M calcium chloride, and the samples were incubated at 37^o^ C for 30 min. After 30 min, the 0.5 M EDTA was added to the reaction to halt enzymatic activity. Each section of skin was homogenized and filtered through a 100 μm filter to yield a single cell suspension. The cell suspension (1 × 10^6^ cells) was washed with PBS and then blocked with 100 μL of a 1:10 dilution of anti-mouse CD16/32 Fc blocking reagent (eBioscience Cat# 14–0161-82) for 15 min at 4^o^ C. Again, the samples were washed with PBS, and 100 μL of a 1:200 dilution of each antibody was added to the samples: CD11b (Biolegend Cat# 101243), CD11c (Biolegend Cat# 117331), F4/80 (Invitrogen Cat# 4323732), MHCII (eBioscience Cat# 47–5321-82), and fixable viability dye (Invitrogen Cat # 4339520). The samples were incubated in antibody for 30 min at 4^o^ C, and then washed with PBS. Each sample was fixed by incubating in 250 μL of a 2.5% formalin solution for 20 min at 4^o^ C, before washing in PBS and suspending the samples in 300 μL of PBS. Appropriate compensation controls and fluorescence minus one samples were prepared alongside the treated samples. The stained cell suspensions were run through an 18-color LSRII flow cytometer (BD Biosciences) at a 35 μL/minute flow rate, for 5 min each. The data was analyzed using FlowJo version 8.8.7 (FlowJo LLC, Ashland, OR). (Example gating scheme in Additional file 1: Figure S4) All flow cytometry data presented are percentages of live cell populations in the skin sections.

### Cytokine/chemokine analysis

A 1 cm × 1 cm piece of each treated skin sample was placed in 400 μL of tissue protein extraction reagent (Thermoscientific Cat# 78510) with a 1:1000 dilution of HALT protease inhibitor cocktail (Thermoscientific Cat3 78,410) added. The skin samples were homogenized and incubated for 15 min at 4^o^ C. The samples were then centrifuged for 5 min at 12,000 RPM to pellet the tissue debris. Protein concentrations were determined using a BCA protein assay (Pierce Cat# 23225). The following cytokines and chemokines were analyzed via a bead based ELISA kit (Millipore Cat# MYCYTOMAG-70 K): MCP-1, TNFα, IP-10, MIP-1α, MIP-2, KC, IL-6, IL-1β. The samples were all analyzed using the Bio-Plex 200 system (Bio-Rad), and at least 50 beads were analyzed per sample.

### Statistics

All statistical analyses were performed with JMP Pro version 13.2.1 (SAS Institute Inc., Cary, NC). A two-way analysis of variance with post-hoc Tukey tests were used when appropriate, unless otherwise specified in the figure legends. *P*-values < 0.05 were considered significant. All data are presented as means ± standard error of the mean (SEM).

## Additional file

Additional file 1: **Table S1.** Carboxylated MWCNT XPS elemental survey. **Figure S1.** Pristine MWCNT display less HaCaT cytotoxicity and more ROS generation than oxidized MWCNT. **Figure S2.** In-house oxidized MWCNT representative XPS plots. **Figure S3.** In-house Oxidized MWCNT TEM images. **Figure S4.** Example flow cytometry gating strategy. (DOCX 2170 kb)

## References

[1] Iijima S (1991). Helical microtubules of graphitic carbon. Nature.

[2] Iijima S, Ichihashi T (1993). Single-shell carbon nanotubes of 1-nm diameter. Nature.

[3] Kingston C, Zepp R, Andrady A, Boverhof D, Fehir R, Hawkins D, Roberts J, Sayre P, Shelton B, Sultan Y (2014). Release characteristics of selected carbon nanotube polymer composites. Carbon.

[4] Maynard AD, Baron PA, Foley M, Shvedova AA, Kisin ER, Castranova V (2004). Exposure to carbon nanotube material: aerosol release during the handling of unrefined single-walled carbon nanotube material. J Toxicol Environ Health Part A.

[5] Lam CW, James JT, McCluskey R, Hunter RL (2004). Pulmonary toxicity of single-wall carbon nanotubes in mice 7 and 90 days after intratracheal instillation. Toxicol Sci.

[6] Shvedova AA, Kisin ER, Mercer R, Murray AR, Johnson VJ, Potapovich AI, Tyurina YY, Gorelik O, Arepalli S, Schwegler-Berry D (2005). Unusual inflammatory and fibrogenic pulmonary responses to single-walled carbon nanotubes in mice. Am J Physiol Lung Cell Mol Physiol.

[7] Mangum JB, Turpin EA, Antao-Menezes A, Cesta MF, Bermudez E, Bonner JC (2006). Single-walled carbon nanotube (SWCNT)-induced interstitial fibrosis in the lungs of rats is associated with increased levels of PDGF mRNA and the formation of unique intercellular carbon structures that bridge alveolar macrophages in situ. Particle Fibre Toxicol.

[8] Mercer RR, Scabilloni J, Wang L, Kisin E, Murray AR, Schwegler-Berry D, Shvedova AA, Castranova V (2008). Alteration of deposition pattern and pulmonary response as a result of improved dispersion of aspirated single-walled carbon nanotubes in a mouse model. Am J Physiol Lung Cell Mol Physiol.

[9] NIOSH (2013). Occupational exposure to carbon nanotubes and nanofibers. Curr Intel Bull.

[10] Dahm MM, Schubauer-Berigan MK, Evans DE, Birch ME, Fernback JE, Deddens JA (2015). Carbon nanotube and nanofiber exposure assessments: an analysis of 14 site visits. Ann Occupat Hygiene.

[11] Dahm MM, Schubauer-Berigan MK, Evans DE, Birch ME, Bertke S, Beard JD, Erdely A, Fernback JE, Mercer RR, Grinshpun SA (2018). Exposure assessments for a cross-sectional epidemiologic study of US carbon nanotube and nanofiber workers. Int J Hyg Environ Health.

[12] Yu C-Y, Hsu Y-W, Chen C-Y (2008). Determination of hand surface area as a percentage of body surface area by 3D anthropometry. Burns.

[13] Strasinger CL, Scheff NN, Wu J, Hinds BJ, Stinchcomb AL (2009). Carbon nanotube membranes for use in the transdermal treatment of nicotine addiction and opioid withdrawal symptoms. Subst Abuse.

[14] Im JS, Bai B, Lee YS (2010). The effect of carbon nanotubes on drug delivery in an electro-sensitive transdermal drug delivery system. Biomaterials.

[15] Kuche K, Maheshwari R, Tambe V, Mak KK, Jogi H, Raval N, Pichika MR, Kumar Tekade R (2018). Carbon nanotubes (CNTs) based advanced dermal therapeutics: current trends and future potential. Nanoscale.

[16] Sun J, Zhao Y, Yang Z, Shen J, Cabrera E, Lertola MJ, Yang W, Zhang D, Benatar A, Castro JM, et al. Highly stretchable and ultrathin Nanopaper composites for epidermal strain sensors. Nanotechnology. 2018;29(35):355304. 10.1088/1361-6528/aacc59 29897348

[17] Oh SY, Hong SY, Jeong YR, Yun J, Park H, Jin SW, Lee G, Oh JH, Lee H, Lee SS (2018). Skin-attachable, stretchable electrochemical sweat sensor for glucose and pH detection. ACS Appl Mater Interfaces.

[18] Liu B, Luo Z, Zhang W, Tu Q, Jin X. Carbon nanotube-based self-adhesive polymer electrodes for wireless long-term recording of electrocardiogram signals. J Biomaterials Sci Polymer Edit. 2016:1–10. 10.1080/09205063.2016.1239951 27659794

[19] He Y, Ming Y, Li W, Li Y, Wu M, Song J, Li X, Liu H. Highly stable and flexible pressure sensors with modified multi-walled carbon nanotube/polymer composites for human monitoring. Sensors (Basel, Switzerland). 2018;18(5). 10.3390/s18051338. 10.3390/s18051338 PMC5982526 29701643

[20] Lima R, Alcaraz-Espinoza JJ, da Silva FAG, de Oliveira HP (2018). Multifunctional wearable electronic textiles using cotton fibers with Polypyrrole and carbon nanotubes. ACS Appl Mater Interfaces.

[21] Cao R, Pu X, Du X, Yang W, Wang J, Guo H, Zhao S, Yuan Z, Zhang C, Li C, et al. Screen-printed washable electronic textiles as self-powered touch/gesture Tribo-sensors for intelligent human-machine interaction. ACS Nano. 2018;12(6):5190-96. 10.1021/acsnano.8b02477 29771494

[22] Sim HJ, Choi C, Kim SH, Kim KM, Lee CJ, Kim YT, Lepro X, Baughman RH, Kim SJ (2016). Stretchable triboelectric Fiber for self-powered kinematic sensing textile. Sci Rep.

[23] Shvedova AA, Castranova V, Kisin ER, Schwegler-Berry D, Murray AR, Gandelsman VZ, Maynard A, Baron P (2003). Exposure to carbon nanotube material: assessment of nanotube cytotoxicity using human keratinocyte cells. J Toxicol Environ Health Part A.

[24] Manna SK, Sarkar S, Barr J, Wise K, Barrera EV, Jejelowo O, Rice-Ficht AC, Ramesh GT (2005). Single-walled carbon nanotube induces oxidative stress and activates nuclear transcription factor-kappaB in human keratinocytes. Nano Lett.

[25] Monteiro-Riviere NA, Nemanich RJ, Inman AO, Wang YY, Riviere JE (2005). Multi-walled carbon nanotube interactions with human epidermal keratinocytes. Toxicol Lett.

[26] Witzmann FA, Monteiro-Riviere NA (2006). Multi-walled carbon nanotube exposure alters protein expression in human keratinocytes. Nanomed.

[27] Patlolla A, Knighten B, Tchounwou P (2010). Multi-walled carbon nanotubes induce cytotoxicity, genotoxicity and apoptosis in normal human dermal fibroblast cells. Ethnicity Dis.

[28] McShan D, Yu H (2014). DNA damage in human skin keratinocytes caused by multiwalled carbon nanotubes with carboxylate functionalization. Toxicol Ind Health.

[29] Kishore AS, Surekha P, Murthy PB (2009). Assessment of the dermal and ocular irritation potential of multi-walled carbon nanotubes by using in vitro and in vivo methods. Toxicol Lett.

[30] Ema M, Matsuda A, Kobayashi N, Naya M, Nakanishi J (2011). Evaluation of dermal and eye irritation and skin sensitization due to carbon nanotubes. Regul Toxicol Pharmacol.

[31] Murray AR, Kisin E, Leonard SS, Young SH, Kommineni C, Kagan VE, Castranova V, Shvedova AA (2009). Oxidative stress and inflammatory response in dermal toxicity of single-walled carbon nanotubes. Toxicology.

[32] Luckhaupt SE, Dahlhamer JM, Ward BW, Sussell AL, Sweeney MH, Sestito JP, Calvert GM (2013). Prevalence of dermatitis in the working population, United States, 2010 National Health Interview Survey. Am J Ind Med.

[33] Landis ET, Davis SA, Taheri A, Feldman SR (2014). Top dermatologic diagnoses by age. Dermatol Online J.

[34] Jatana S, Palmer BC, Phelan SJ, DeLouise LA (2017). Immunomodulatory effects of nanoparticles on skin allergy. Sci Rep.

[35] Datsyuk V, Kalyva M, Papagelis K, Parthenios J, Tasis D, Siokou A, Kallitsis I, Galiotis C (2008). Chemical oxidation of multiwalled carbon nanotubes. Carbon.

[36] Welzel J, Metker C, Wolff HH, Wilhelm KP (1998). SLS-irritated human skin shows no correlation between degree of proliferation and TEWL increase. Arch Dermatol Res.

[37] Bonneville M, Chavagnac C, Vocanson M, Rozieres A, Benetiere J, Pernet I, Denis A, Nicolas JF, Hennino A (2007). Skin contact irritation conditions the development and severity of allergic contact dermatitis. J Invest Dermatol.

[38] Lee JJ, Protheroe CA, Luo H, Ochkur SI, Scott GD, Zellner KR, Raish RJ, Dahl MV, Vega ML, Conley O (2015). Eosinophil-dependent skin innervation and itching following contact toxicant exposure in mice. J Allergy Clin Immunol.

[39] Honda T, Egawa G, Grabbe S, Kabashima K (2013). Update of immune events in the murine contact hypersensitivity model: toward the understanding of allergic contact dermatitis. J Investig Dermatol.

[40] Kim BS, Miyagawa F, Cho YH, Bennett CL, Clausen BE, Katz SI (2009). Keratinocytes function as accessory cells for presentation of endogenous antigen expressed in the epidermis. J Invest Dermatol.

[41] Grone A (2002). Keratinocytes and cytokines. Vet Immunol Immunopathol.

[42] Dilulio NA, Engeman T, Armstrong D, Tannenbaum C, Hamilton TA, Fairchild RL (1999). Groalpha-mediated recruitment of neutrophils is required for elicitation of contact hypersensitivity. Eur J Immunol.

[43] Biedermann T (2000). Mast Cells Control Neutrophil Recruitment during T Cell–Mediated Delayed-Type Hypersensitivity Reactions through Tumor Necrosis Factor and Macrophage Inflammatory Protein 2. J Exp Med.

[44] Lv J, Huang Y, Zhu S, Yang G, Zhang Y, Leng J, Bo J, Liu D (2012). MCP-1-induced histamine release from mast cells is associated with development of interstitial cystitis/bladder pain syndrome in rat models. Mediat Inflamm.

[45] Dufour JH, Dziejman M, Liu MT, Leung JH, Lane TE, Luster AD (2002). IFN-gamma-inducible protein 10 (IP-10; CXCL10)-deficient mice reveal a role for IP-10 in effector T cell generation and trafficking. J Immunol.

[46] Reichel CA, Rehberg M, Lerchenberger M, Berberich N, Bihari P, Khandoga AG, Zahler S, Krombach F (2009). Ccl2 and Ccl3 mediate neutrophil recruitment via induction of protein synthesis and generation of lipid mediators. Arterioscler Thromb Vasc Biol.

[47] Christensen AD, Haase C (2012). Immunological mechanisms of contact hypersensitivity in mice. APMIS.

[48] Watanabe H, Gaide O, Pétrilli V, Martinon F, Contassot E, Roques S, Kummer JA, Tschopp J, French LE (2007). Activation of the IL-1β-processing Inflammasome is involved in contact hypersensitivity. J Investig Dermatol.

[49] Dudeck A, Dudeck J, Scholten J, Petzold A, Surianarayanan S, Köhler A, Peschke K, Vöhringer D, Waskow C, Krieg T (2011). Mast cells are key promoters of contact allergy that mediate the adjuvant effects of Haptens. Immunity.

[50] Weber FC, Nemeth T, Csepregi JZ, Dudeck A, Roers A, Ozsvari B, Oswald E, Puskas LG, Jakob T, Mocsai A (2015). Neutrophils are required for both the sensitization and elicitation phase of contact hypersensitivity. J Exp Med.

[51] Gocinski BL, Tigelaar RE (1990). Roles of CD4+ and CD8+ T cells in murine contact sensitivity revealed by in vivo monoclonal antibody depletion. J Immunol.

[52] Miyazaki D, Nakamura T, Toda M, Cheung-Chau KW, Richardson RM, Ono SJ (2005). Macrophage inflammatory protein–1α as a costimulatory signal for mast cell–mediated immediate hypersensitivity reactions. J Clin Invest.

[53] Askenase PW, Tsuji RF (2000). B-1 B cell IgM antibody initiates T cell elicitation of contact sensitivity. Curr Top Microbiol Immunol.

[54] Lunderius-Andersson C, Enoksson M, Nilsson G. Mast cells respond to cell injury through the recognition of IL-33. Front Immunol. 2012;3:82. 10.3389/fimmu.2012.00082 PMC3342375 22566963

[55] Dawicki W, Marshall JS (2007). New and emerging roles for mast cells in host defence. Curr Opin Immunol.

[56] Christy AL, Walker ME, Hessner MJ, Brown MA (2013). Mast cell activation and neutrophil recruitment promotes early and robust inflammation in the meninges in EAE. J Autoimmun.

[57] Chen R, Ning G, Zhao M-L, Fleming MG, Diaz LA, Werb Z, Liu Z (2001). Mast cells play a key role in neutrophil recruitment in experimental bullous pemphigoid. J Clin Invest.

[58] Henry CM, Sullivan GP, Clancy DM, Afonina IS, Kulms D, Martin SJ (2016). Neutrophil-derived proteases escalate inflammation through activation of IL-36 family cytokines. Cell Rep.

[59] Mócsai A (2013). Diverse novel functions of neutrophils in immunity, inflammation, and beyond. J Exp Med.

[60] Engeman T, Gorbachev AV, Kish DD, Fairchild RL (2004). The intensity of neutrophil infiltration controls the number of antigen-primed CD8 T cells recruited into cutaneous antigen challenge sites. J Leukoc Biol.

[61] Wei JCJ, Edwards GA, Martin DJ, Huang H, Crichton ML, Kendall MAF (2017). Allometric scaling of skin thickness, elasticity, viscoelasticity to mass for micro-medical device translation: from mice, rats, rabbits, pigs to humans. Sci Rep.

[62] Segre JA (2006). Epidermal barrier formation and recovery in skin disorders. J Clin Invest.

[63] Inoue K, Koike E, Yanagisawa R, Hirano S, Nishikawa M, Takano H (2009). Effects of multi-walled carbon nanotubes on a murine allergic airway inflammation model. Toxicol Appl Pharmacol.

[64] Ryman-Rasmussen JP, Tewksbury EW, Moss OR, Cesta MF, Wong BA, Bonner JC (2009). Inhaled multiwalled carbon nanotubes potentiate airway fibrosis in murine allergic asthma. Am J Respir Cell Mol Biol.

[65] Beamer CA, Girtsman TA, Seaver BP, Finsaas KJ, Migliaccio CT, Perry VK, Rottman JB, Smith DE, Holian A. IL-33 mediates multi-walled carbon nanotube (MWCNT)-induced airway hyper-reactivity via the mobilization of innate helper cells in the lung. Nanotoxicology. 2013, 7(6):1070–81. 10.3109/17435390.2012.702230 PMC4080677 22686327

[66] Hussain S, Sangtian S, Anderson SM, Snyder RJ, Marshburn JD, Rice AB, Bonner JC, Garantziotis S (2014). Inflammasome activation in airway epithelial cells after multi-walled carbon nanotube exposure mediates a profibrotic response in lung fibroblasts. Particle Fibre Toxicol.

[67] Ronzani C, Casset A, Pons F (2014). Exposure to multi-walled carbon nanotubes results in aggravation of airway inflammation and remodeling and in increased production of epithelium-derived innate cytokines in a mouse model of asthma. Arch Toxicol.

[68] Sager TM, Wolfarth MW, Andrew M, Hubbs A, Friend S, Chen TH, Porter DW, Wu N, Yang F, Hamilton RF (2014). Effect of multi-walled carbon nanotube surface modification on bioactivity in the C57BL/6 mouse model. Nanotoxicology.

[69] Silva RM, Doudrick K, Franzi LM, TeeSy C, Anderson DS, Wu Z, Mitra S, Vu V, Dutrow G, Evans JE (2014). Instillation versus inhalation of multiwalled carbon nanotubes: exposure-related health effects, clearance, and the role of particle characteristics. ACS Nano.

[70] Sayes CM, Liang F, Hudson JL, Mendez J, Guo W, Beach JM, Moore VC, Doyle CD, West JL, Billups WE (2006). Functionalization density dependence of single-walled carbon nanotubes cytotoxicity in vitro. Toxicol Lett.

[71] Hamilton RF, Wu Z, Mitra S, Shaw PK, Holian A (2013). Effect of MWCNT size, carboxylation, and purification on in vitro and in vivo toxicity, inflammation and lung pathology. Particle Fibre Toxicol.

[72] Singh RP, Das M, Thakare V, Jain S (2012). Functionalization density dependent toxicity of oxidized multiwalled carbon nanotubes in a murine macrophage cell line. Chem Res Toxicol.

[73] Ago H, Kugler T, Cacialli F, Salaneck WR, Shaffer MSP, Windle AH, Friend RH (1999). Work functions and surface functional groups of multiwall carbon nanotubes. J Phys Chem B.

[74] Boukamp P, Petrussevska RT, Breitkreutz D, Hornung J, Markham A, Fusenig NE (1988). Normal keratinization in a spontaneously immortalized aneuploid human keratinocyte cell line. J Cell Biol.

[75] Schneider CA, Rasband WS, Eliceiri KW (2012). NIH image to ImageJ: 25 years of image analysis. Nat Methods.

