# Supplementary material for: Multi-walled carbon nanotube oxidation dependent keratinocyte cytotoxicity and skin inflammation
Source: Part Fibre Toxicol. 2019 Jan 8;16:3. doi: 10.1186/s12989-018-0285-x (PMC6323751; doi:10.1186/s12989-018-0285-x)
Supplement: Supplementary file 1 — Table S1. Carboxylated MWCNT XPS elemental survey. Figure S1. Pristine MWCNT display less HaCaT cytotoxicity and more ROS generation than oxidized MWCNT. Figure S2. In-house oxidized MWCNT representative XPS plots. Figure S3. In-house Oxidized MWCNT TEM images. Figure S4. Example flow cytometry gating strategy. (DOCX 2170 kb) [file 12989_2018_285_MOESM1_ESM.docx]

**Supplemental Material**

**Multi-walled Carbon Nanotube Oxidation Dependent Keratinocyte Cytotoxicity and Skin Inflammation**

Brian C. Palmer^a^, Sarah J. Phelan^a^, Lisa A. DeLouise^a,b,c^

^a^Department of Environmental Medicine, University of Rochester Medical Center, New York, USA

^b^Department of Biomedical Engineering, University of Rochester, Rochester, New York, USA.

^c^Department of Dermatology, University of Rochester Medical Center, Rochester, New York, USA.

Lisa A. DeLouise

University of Rochester Medical Center

School of Medicine and Dentistry

601 Elmwood Avenue, Box 697

Rochester, NY-14642

Tel: (585) 275-1810

E-mail: [Lisa_DeLouise@urmc.rochester.edu](mailto:Lisa_DeLouise@urmc.rochester.edu)

Additional file 1: Table S1. Carboxylated MWCNT XPS elemental survey.

| **MWCNT** | **Carbon (%)** | **Oxygen (%)** | **Sodium (%)** | **Chlorine (%)** | **Magnesium (%)** |
| --- | --- | --- | --- | --- | --- |
| Low COOH | 79.76 | 13.37 | 2.04 | 4.83 | - |
| High COOH | 27.97 | 12.84 | 10.82 | 16.98 | 11.39 |
| Pristine | 99.41 | 0.59 | - | - | - |
| 1.5 Hour  Oxidation | 41.08 | 16.36 | 13.96 | 28.60 | - |
| 3 Hour Oxidation | 32.57 | 12.55 | 13.90 | 40.98 | - |
| 6 Hour Oxidation | 46.44 | 27.69 | 12.83 | 13.05 | - |
| 12 Hour Oxidation | 51.41 | 23.09 | 11.22 | 14.27 | - |

The suspensions of each nanoparticle were dried onto silicon wafers, and the elemental survey was examined via XPS. The complete list of elements detected for each sample are listed in the table, and no transition metal catalyst was detected in any sample.

Additional file 1: Figure S1. Pristine MWCNT display less HaCaT cytotoxicity and more ROS generation than oxidized MWCNT.


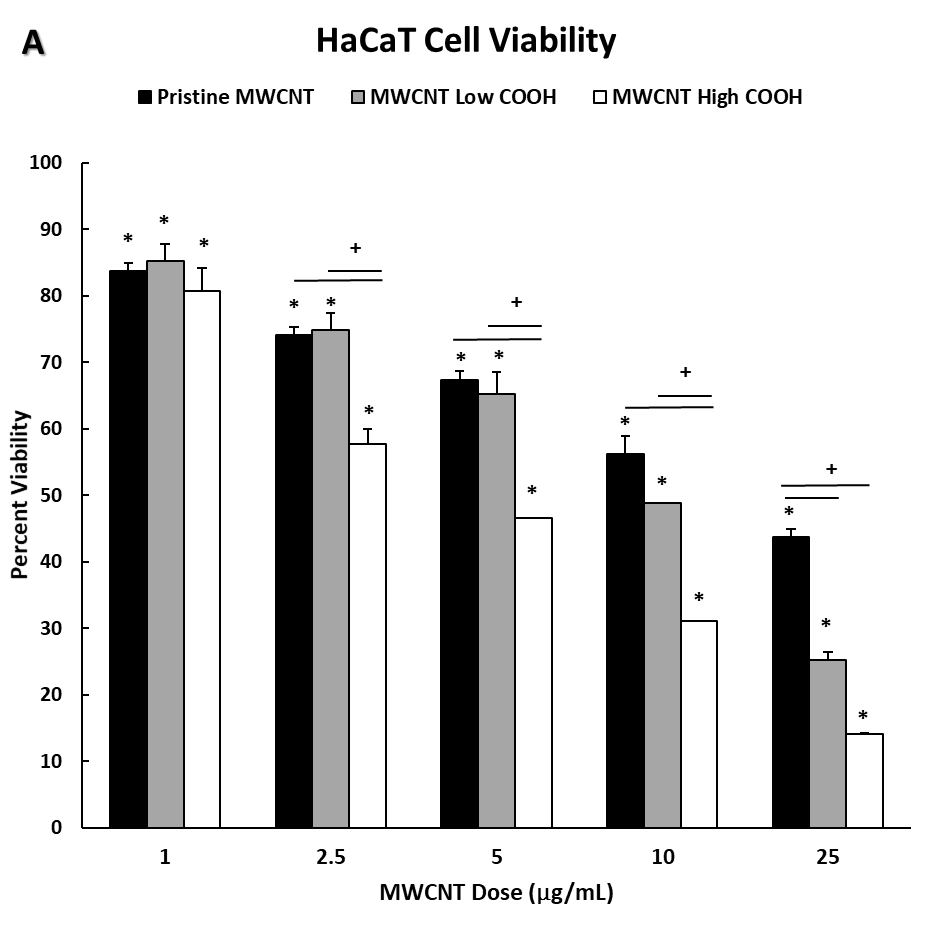


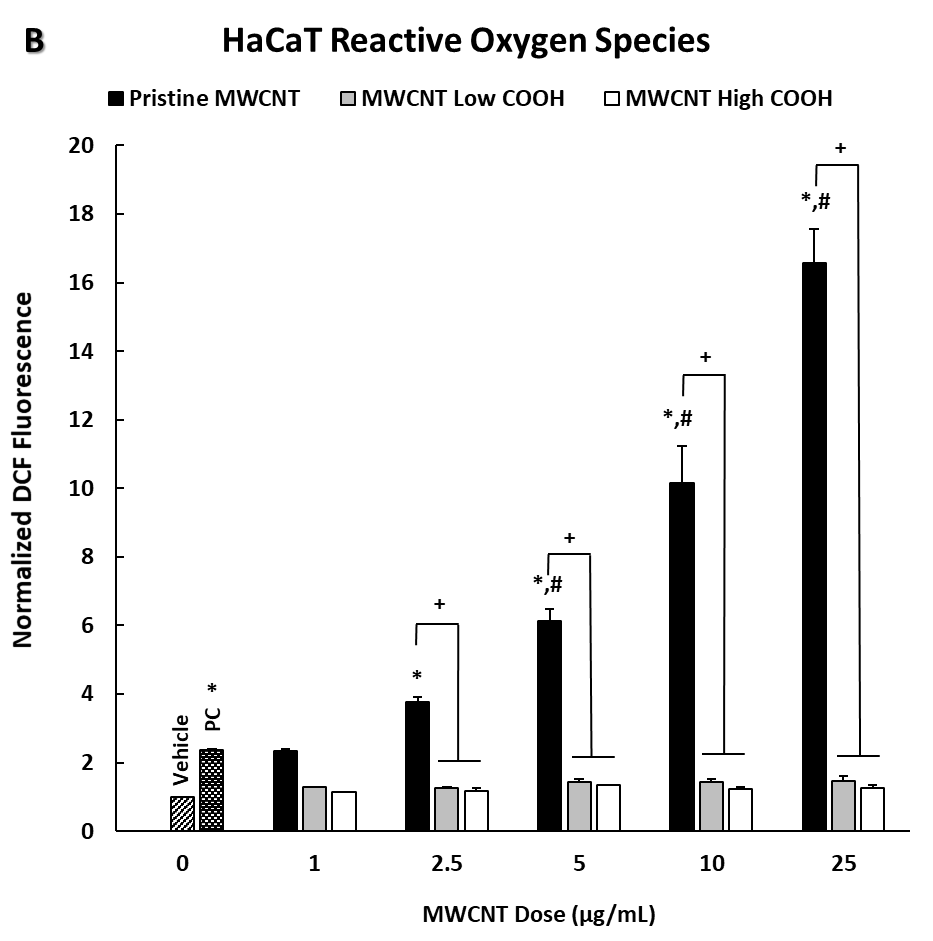


An immortalized human keratinocyte cell line (HaCaTs) were grown to 70% confluency in DMEM supplemented with 10% FBS and 1% P/S. The cells were treated with 0-25 μg/mL of pristine, low-COOH, or high-COOH MWCNT, for 24 hours. Cytotoxicity was measured using the cell-titer glo luminescence based ATP assay (A). ROS was measured by DCF-DA fluorescence in a plate based assay (B). Graphs represent means (+/- SEM), N=3. Significance is defined at a p-value < 0.05. * indicate significance compared to vehicle, # indicate significance compared to the 100 μM hydrogen peroxide positive control, and + indicates significance within groups.

Additional file 1: Figure S2. In-house oxidized MWCNT representative XPS plots.


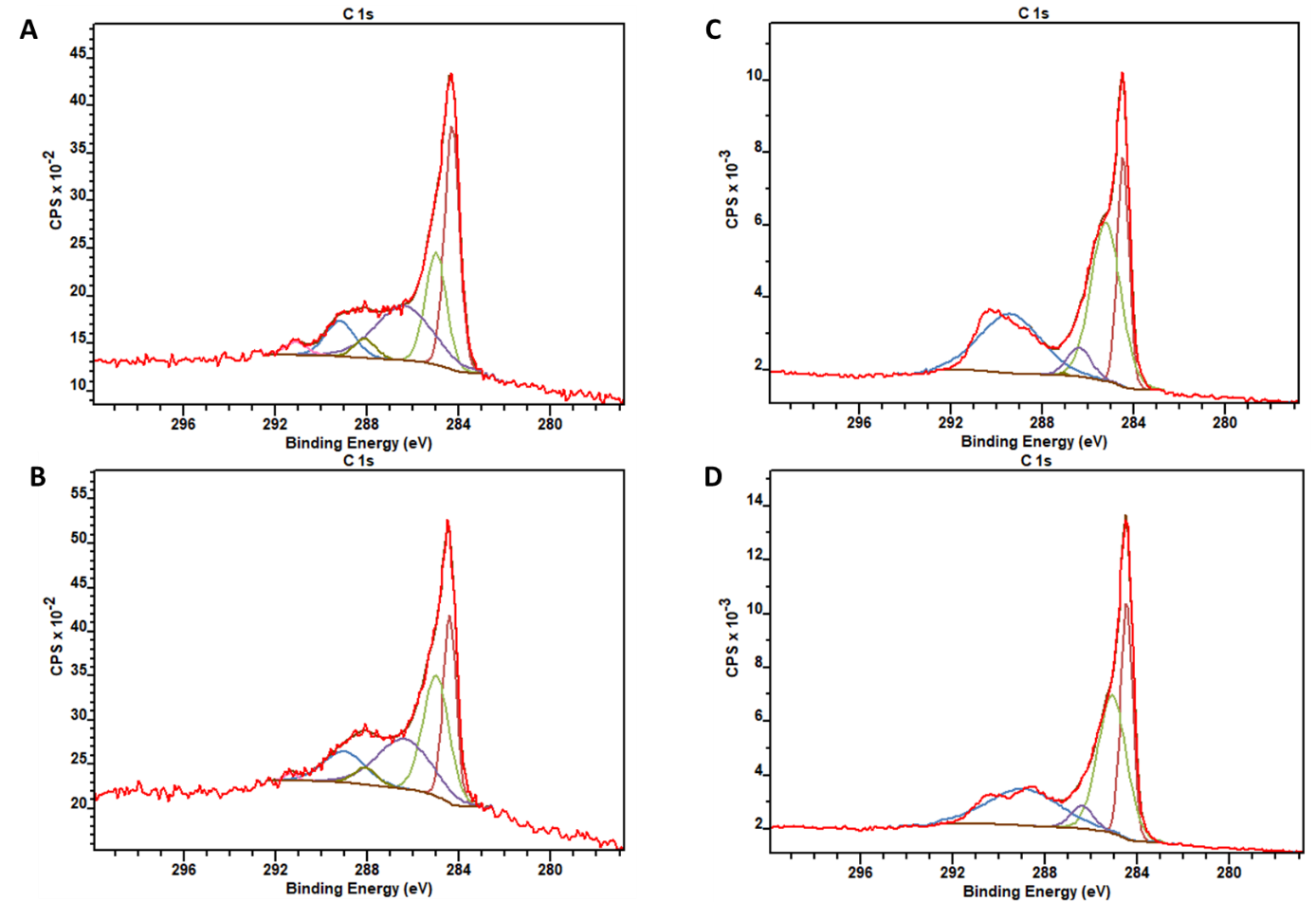


The suspensions of each nanoparticle were dried onto silicon wafers and examined via XPS. The representative plots of the C 1s region for the 1.5 hour oxidized MWCNT (A), 3 hour oxidized MWCNT (B), 6 hour oxidized MWCNT (C), and 12 hour oxidized MWCNT (D) display the differences in carbon oxidation.

Additional file 1: Figure S3. In-house Oxidized MWCNT TEM images.


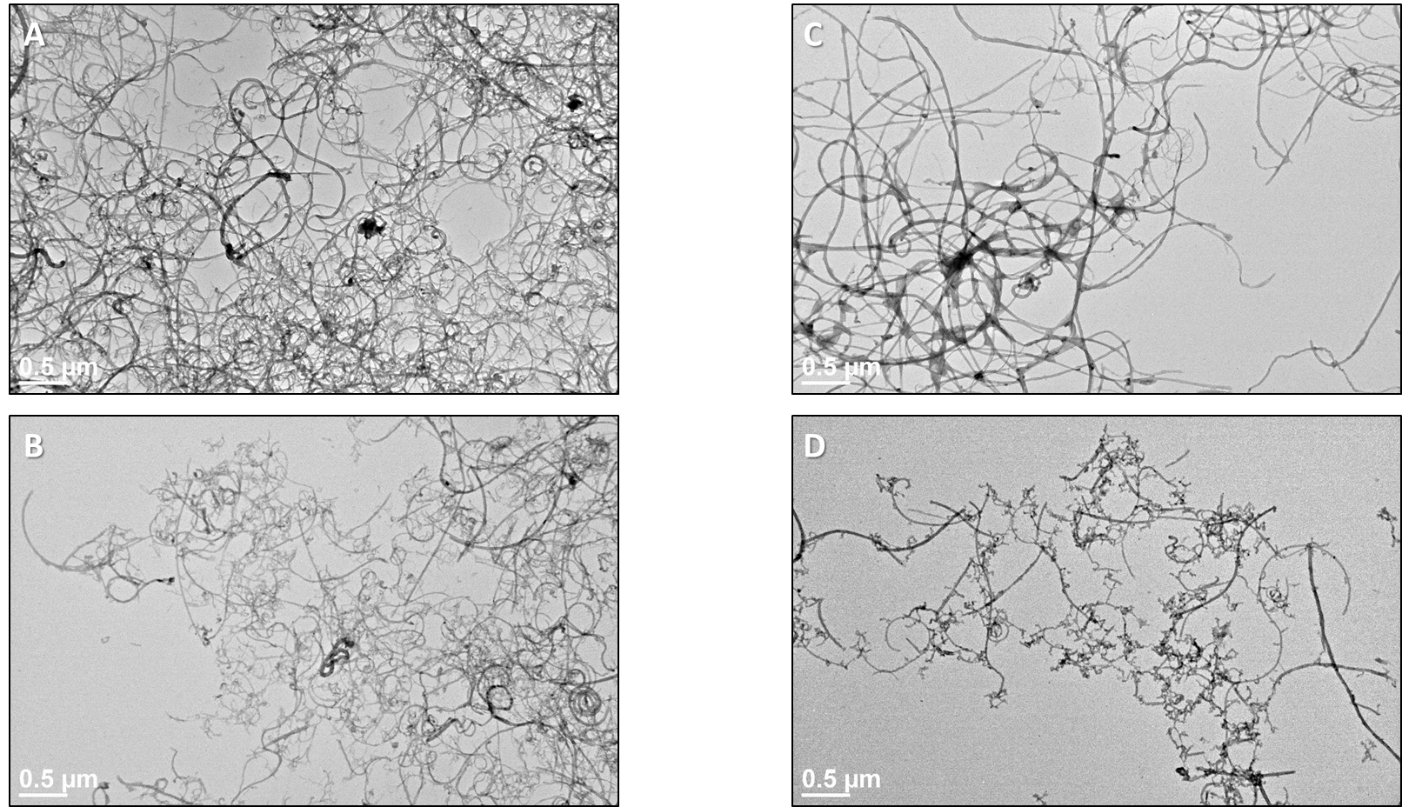


The 1 mg/mL suspensions of each nanoparticle were dried onto TEM grids for 30 seconds, and representative TEM images of the 1.5 hour oxidized MWCNT (A), 3 hour oxidized MWCNT (B), 6 hour oxidized MWCNT (C), and 12 hour oxidized MWCNT (D) display the general structure of each nanoparticle.


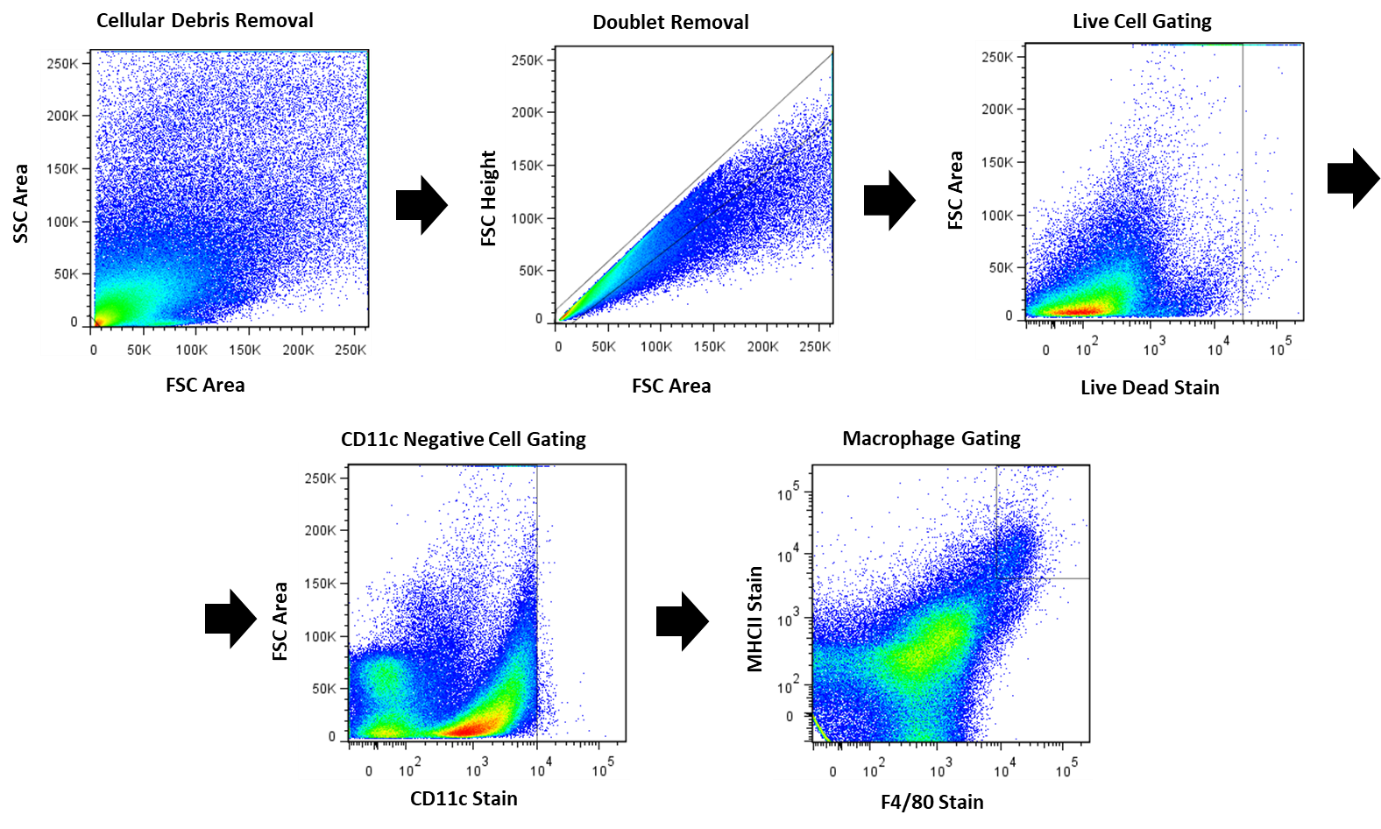
Additional file 1: Figure S4. Example flow cytometry gating strategy.

For all flow cytometry samples, the cell debris was removed by gating out events with low side and forward scatter. Doublet events were removed by gating on the forward scatter height and area. Only cells that excluded the dead cell stain were included in the gating. As an example, to gate macrophage cells, the cells negative for CD11c were gated on before gating cells high in MHCII and F4/80 staining.
